# The A(ffect) B(ehavior) C(ognition) D(ecision) of parasocial relationships: A pilot study on the psychometric properties of the Multidimensional Measure of Parasocial Relationships (MMPR)

**DOI:** 10.1016/j.heliyon.2022.e10779

**Published:** 2022-09-30

**Authors:** Danilo Garcia, Elina Björk, Maryam Kazemitabar

**Affiliations:** a Department of Behavioral Sciences and Learning, Linköping University, Linköping, Sweden; b Promotion of Health and Innovation (PHI) Lab, International Network for Well-Being, Sweden; c Centre for Ethics, Law and Mental Health (CELAM), University of Gothenburg, Gothenburg, Sweden; d Department of Psychology, University of Gothenburg, Gothenburg, Sweden; e Department of Psychology, Lund University, Lund, Sweden; f Promotion of Health and Innovation (PHI) Lab, International Network for Well-Being, USA; g Yale University School of Public Health, New Haven, Connecticut, USA

**Keywords:** Classical test theory, Parasocial interactions, Parasocial relationships, Self-esteem, Social comparison orientation, Social media

## Abstract

The aim of this pilot study was to preliminary test the psychometric properties of the Multidimensional Measure of Parasocial Relationships (MMPR), a self-report that assess people’s attitude (*affect*, *cognition*, and *behavior*) towards social media figures and to what extent people perceive that media figures influence their daily life *decisions* (e.g., consumption, exercise, nutrition). In short, the MMPR measures how and to what extent people are committed to such one-sided relationships and interactions through social media platforms. Besides factor structural analyses (four different models) and internal consistency, we also tested the MMPR’s concurrent validity by investigating if, as hypothesized, the association between commitment to parasocial relationships and self-esteem is mediated by its positive association to social comparison. Participants (*N* = 259) answered to the MMPR, the Iowa-Netherlands Comparison Orientation Measure, and the Rosenberg Self-Esteem Scale. As expected, the MMPR loaded in four dimensions and had good internal consistency (e.g., *Cronbach’s Alphas* were between .66-.75 for the four dimensions and .85 for the whole measure). The bifactor model with correlated factors had the best fit indexes (*CFI* = .95, *RMSEA* = .07). Moreover, the direct effect of MMPR was positive on social comparison (*β* = .18, *p* < .01), the direct effect of social comparison on self-esteem was negative (*β* = -.51, *p* < .001), and the indirect effect of MMPR on self-esteem was negative (*β* = -.09, *p* < .01). In sum, our results suggest that parasocial relationships through social media platforms consist of four necessary and correlated dimensions (A: Affective; B: Behavioral; C: Cognitive; and D: Decisional). Moreover, the MMPR successfully assessed that high level of commitment with parasocial relationships are positively associated with the tendency to compare oneself to others, which in turn leads to low levels of self-esteem. Hence, the MMPR has sound psychometric properties and is a good candidate for further analyses.

## 1 Introduction

During the 21^st^ century, we are more than ever exposed to celebrities and media figures who through social media platforms might influence our way of thinking, feeling, and living. Such one-sided relationships and interactions with celebrities and media figures are defined as parasocial in nature [1, 2]. In this context, people’s commitment to parasocial relationships is expected to be positively associated with social comparison, which in turn might have detrimental effects on our identity formation, self-esteem, and mental health [3, 4, 5, 6]. In order to understand this phenomenon, researchers have developed different single and multidimensional measures, such as, the Celebrity-Persona Parasocial Interaction Scale [7]; the Audience Persona Interaction Scale with four dimensions addressing identification with favorite character, interest in favorite character, group identification/interaction, and favorite character’s problem solving ability [8]; the Experience of Parasocial Interaction Scale, which measures how people perceive parasocial interaction experiences [2], and the Parasocial Relationship with Political Figures Scale, which assesses ordinary people’s emotional bonding with political figures [9]. However, one common confusion in the literature is the actual distinction between two related concepts: parasocial relationship and parasocial interaction.

Parasocial relationships as a concept refers to “a lingering sense of intimacy and connectedness with media personalities”; parasocial interactions refers to “illusionary give-and-take with media figures” [10]. While the former requires that the sense of intimacy to a media figure is kept and prolonged beyond an interaction (e.g., reading the media figure’s Instagram account), a parasocial interaction is the single experience with an actual one-to-one moment or at least the feeling that the media figure is aware of the viewer/reader [10]. In other words, people can have a parasocial interaction without having a parasocial relationship and vice versa [10]. Nevertheless, repeated parasocial interactions with a media figure have been suggested to increase the sense of having a parasocial relationship with the media figure [11]. One way or another, being dedicated to a parasocial relationship (i.e., one’s level of commitment) through a social media platform consists of one type of behavior that involves the “follower” to interact with the media figure by, for example, “liking” her/his posts. This kind of parasocial interaction might involve the sense of a give-and-take moment with the media figure; but the media figure is most of the times posting her/his thoughts and pictures to an “Army” of followers. Importantly, “likes” in social media, which allows social media users to interact with updates and show their approval of what the media figure is sharing, are highly predictive of the user’s future behavior and even her own self-reported personality [12]. In other words, commitment to parasocial relationships through social media might influence several dimensions of people’s lives. Here we argue that commitment to parasocial relationships through social media platforms needs to be understood as people’s attitude towards the social media figure, but also considering how much the individual feels influenced in her/his daily life decisions (e.g., consumption, exercise, nutrition) by the media figure.

In this line of thinking, we have developed the Multidimensional Measure of Parasocial Relationships (MMPR), which is primarily based on the multidimensional model of attitude [13]. In accordance with this classical view of the concept of attitude formation, the MMPR operationalizes peoples’ feelings (A: Affective Dimension) and thoughts (C: Cognitive Dimension) about a social media figure they “follow”, and people’s way of interacting with the media figure through social media platforms (B: Behavioral Dimension). The MMPR also includes the extended influence of attitudes within a parasocial relationship by considering the perceived influence that the media figures have on people’s daily life decisions (D: Decisional Dimension). In other words, the MMPR measures how and to what extent people are committed to parasocial relationships.

The Affective dimension of the MMPR assess the immediate and prolonged emotional responses to a social media figure and their shared content on social media platforms (e.g., posts). This includes people’s feelings of perceived connectedness, inspiration, and emotional commitment to the social media figure (e.g., “I experience a feeling of connectedness with the media figure through his/her posts on social media”). The Behavioral dimension measures behavioral responses directly tied to the parasocial experience (i.e., reading/viewing content posted by the social media figure). In other words, it refers to people’s actual behavior in a parasocial relationship operationalized as “liking”, sharing and/or commenting the content on the social media figure’s platforms (e.g., “I often forward the media figure's posts to my friends or share them on my own online feeds”). The Cognitive dimension assess people’s thoughts about the media figure (i.e., attributed characteristics and values) and perceived meaning of the content on social media platforms (e.g., “I think that the media figure represents values that are important to me”). Finally, the MMPR includes a Decisional dimension which measures to what extent people experience that the media figure, through her/his shared content on social media, influence their own daily life decisions (e.g., “I happily follow different tips and advice that the media figure shares because I feel I can trust his/her knowledge about these things”). See Table 1 for the MMPR’s Instructions, Items, Rating Scale, and Coding.

**Table 1:** The Multidimensional Measure of Parasocial Relationships (MMPR).

	Item	Swedish	English
**Instructions**	-	Detta formulär består av ett antal påståenden som handlar om dina attityder, det vill säga tankar, känslor och beteenden, som berör en specifik mediaprofil i sociala medier. Innan du anger dina svar funderar du ut en specifik mediaprofil att utgå från, förslagsvis den du följer mest. *Det kan* vara en influencer, youtuber eller annan profil inom exempelvis livsstil, träning, sport, gaming, kost eller mode. *Det viktiga* är att det är en mediaprofil som du inte har någon relation till i verkliga livet. Vänligen ange i vilken grad du instämmer eller tar avstånd från följande påståenden:	This questionnaire contains statements about your attitudes, that is, your thoughts, feelings, and behavior regarding a specific media figure in social media. Before giving your answers, please think of a specific media figure, preferably the one that you follow the most. It can be an influencer, a youtuber or a media figure within, for example, lifestyle, exercise, sports, gaming, nutrition, or fashion. The most important thing is that you don’t have a relation with this person in real life. Please answer to which degree you agree or disagree to the following statements:
**Affective Dimension**	A1	Jag upplever en känsla av samhörighet med mediaprofilen genom hens inlägg i sociala medier	I experience a feeling of connectedness with the media figure through his/her posts on social media.
	A2	Jag upplever att jag blir känslomässigt engagerad när mediaprofilen delar med sig av mer privat information om sig själv (tex större livshändelser)	I experience that I get emotionally engaged when the media figure shares more private information about himself/herself (e.g., bigger life events).
	A3R	Jag känner inte att jag personligen kan relatera till innehållet i mediaprofilens inlägg	I don’t feel like I personally can relate to the content in the media figure's posts.
	A4	Jag känner att jag ofta blir inspirerad av mediaprofilens inlägg	I often feel that I get inspired by the media figure's posts.
**Behavior Dimension**	B1	Jag ”gillar”/”likear” alltid mediaprofilens inlägg i sociala medier	I always “like” the media figure’s posts on social media.
	B2	Jag kommenterar ofta mediaprofilens inlägg i anslutna kommentarsfält	I often comment on the media figure's posts in the comment field.
	B3	Jag delar ofta mediaprofilens inlägg vidare till vänner eller i mina egna flöden i sociala medier	I often forward the media figure's posts to my friends or share them on my own online feeds.
	B4R	Jag kollar mest mediaprofilens uppdateringar och är inte särskilt aktiv med att gilla, dela eller kommentera	I mostly just check the medial figure's updates and aren’t that active with liking, sharing, or commenting.
**Cognitive Dimension**	C1	Jag tycker att mediaprofilen representerar värderingar som är viktiga för mig	I think that the media figure represents values that are important to me.
	C2R	Jag anser inte att mediaprofilen framhäver sig själv på ett verklighetstroget sätt i sociala medier	I don’t think that the media figure portrays himself/herself in an authentic way on social media.
	C3	Jag ser positivt på det mesta som mediaprofilen delar med sig av i sociala medier	I see positive on most of what the media figure shares on social media.
	C4	Mediaprofilen verkar vara en genuin person som jag skulle komma bra överens med i verkligheten	The media figure seems to be a genuine person that I would get along with in real life.
**Decisional Dimension**	D1	Jag föredrar sådant som mediaprofilen marknadsför (produkter, kostråd, träningstips etc) framför liknande saker som marknadsförs på andra platser	I prefer things that the media figure is marketing (e.g., products, nutrition advice, training advice, etc.) before similar things that are marketed in other places.
	D2	Mediaprofilens inlägg gör mig ofta inspirerad att genomföra förändringar i mitt eget liv	The media figure's posts often inspire me to make changes in my own life.
	D3R	Jag köper aldrig produkter som mediaprofilen marknadsför eller tipsar om i sociala medier	I never buy products that the media figure is marketing or giving advice about on social media.
	D4	Jag följer gärna olika tips och råd som mediaprofilen delar med sig av eftersom jag känner att jag kan förlita mig på hens kunskap om dessa saker	I happily follow different tips and advice that the media figure shares because I feel I can trust his/her knowledge about these things.
	D5	Det händer ofta att jag i samtal med andra personer i min vardag, framhäver saker som mediaprofilen nämnt i sina inlägg i sociala medier	It often happens that I, in conversations with other people in my everyday life, point out things that the media figure has mentioned in his/her posts on social media.
	D6	Det händer att mediaprofilens inlägg bidrar till att jag på något vis förändrar mina levnadsvanor (tex klädsel, kostvana, träningsrutin, utseende etc)	It happens that the media figure's posts contribute to, that I in some way change my life habits (e.g., cloths, diet, training routine, looks etc.).

Note: The statements are assessed using the following 4-point Likert scale [Swedish in brackets]: 1 = Totally disagree [Tar avstånd helt], 2 = Partly disagree [Tar delvis avstånd], 3 = Parly agree [Instämmer delvis], 4 = Totally agree [Instämmer helt]. R = Reversed item. Translation to English by Patricia Rosenberg, Johanna Ekberg, and Danilo Garcia. For any use, research or commercial, please contact elina.bjork@icloud.com and danilo.garcia@icloud.com.

In the present pilot study, we aimed to preliminary investigate the psychometric properties of the MMPR by testing its factor structure (i.e., Exploratory Factor Analysis and Confirmatory Factor Analysis), internal consistency (i.e., Cronbach’s Alpha and correlations between factors/dimensions), and its concurrent validity (i.e., by testing if social comparison mediates the relationship between commitment to parasocial relationships and self-esteem).

## 2 Method

### Ethical statement

The data in this study was part of a thesis in psychology [14] in which participation was voluntary, anonymous, no personal data was collected, and the data was not collected for commercial or other non-scientific purposes. After consulting the Swedish law (2003: 460, section 2) concerning research involving humans, we arrived at the conclusion that only informed verbal consent from participants was required and that no ethical approval was necessary for conducting the study.

### 2.1 Participants

A convenient sample, collected through a social media platform, comprising 259 participants (207 women, 51 men, 1 other) with an age mean of 25.30 (*SD* = 5.15) answered to the MMPR, the Iowa-Netherlands Comparison Orientation Measure [15], and the Rosenberg Self-Esteem Scale [16].

### 2.2 Measures

#### 2.2.1 Commitment to parasocial relationships

The MMPR operationalizes peoples’ feelings (A: Affective Dimension) and thoughts (C: Cognitive Dimension) about a social media figure they “follow”, and people’s way of interacting with the media figure through social media platforms (B: Behavioral Dimension). The MMPR also includes a fourth dimension that operationalizes to what extent people perceive that the media figure influences their daily life decisions (D: Decisional Dimension). The MMPR consist of 18 statements that are assessed using a 4-point Likert scale (1 = *Totally Disagree*, 4 = *Totally Agree*). See Table 1.

#### 2.2.2 Social comparison

The Iowa-Netherlands Comparison Orientation Measure [15] consist of 11 statements about people’s self-comparisons with others (e.g., “I always pay a lot of attention to how I do things compared with how others do things”). It uses a 5-point scale ranging from “*Strongly disagree*” to “*Strongly agree*”.

#### 2.2.3 Self-esteem

The Rosenberg Self-Esteem Scale [16] consists of 10 statements (e.g., “I feel that I'm a person of worth, at least on an equal plane with others”) that focus on people’s general feelings toward themselves using a 4-point Likert scale (1 = not agree at all, 4 = agree completely).

### 2.3 Statistical procedure

In order to test the factor structure of the MMPR, we first conducted an Exploratory Factor Analysis (EFA). We then tested different factorial models for the MMPR using Confirmatory Factor Analysis (CFA). We analyzed the MMPR’s internal consistency by calculating *Cronbach’s alphas* for each dimension and by calculating the *Pearson correlation coefficients* between each dimension. Finally, we used Structural Equation Modeling (SEM) to test if the relationship between participants' total MMPR-score and self-esteem was mediated by individuals’ social comparison orientation (i.e., concurrent validity). In other words, this last analysis aimed to test earlier expectations derived from past research [3, 4, 5, 6]—that people’s commitment to parasocial relationships is positively associated with social comparison, which in turn has detrimental effects on our self-esteem.

## 3 Results

### 3.1 Exploratory Factor Analysis (EFA)

The EFA resulted in a *KMO* = .85, which means that the sample size was sufficient for further analyses using the whole sample. In addition, Bartlett’s Test of Sphericity also showed that the items were correlated and suitable for conducting Principal Component Analysis (*χ*^*2*^ = 1573.94, *df* = 153, *p* < .001). The results of the Principal Component Analysis with varimax rotation showed that the MMPR had four components with *eigenvalues* higher than 1 and a cumulative of 56.4% of the total variance. The loadings for the items ranged from .53 (item A3) to .80 (item D6). Since all the items had loadings higher than .50, there was no need to remove any item for subsequent analyses [17, 18]. We show the loadings for each item within their respective factor in Table 2. In Table 3 we show the component transformation matrix, in which the correlations among components are displayed prior and after rotation.

**Table 2:** Rotated Item loadings in Principal Component Analysis with Varimax rotation.

	Items	Components			
		1	2	3	4
**Affective Dimension**	A1	.649	.337	.083	-.014
	A2	.492	.224	.182	-.007
	A3R	.537	.016	.054	.255
	A4	.538	.585	.024	.110
**Behavioral Dimension**	B1	.456	.193	.557	.198
	B2	.044	.197	.742	-.135
	B3	-.097	.186	.734	-.166
	B4R	.057	-.116	.735	.362
**Cognitive Dimension**	C1	.680	.224	.015	.105
	C3	.740	.172	-.045	-.175
	C4	.778	.215	.004	-.094
	C2R	.552	-.245	-.136	.382
**Decisional Dimension**	D1	.147	.630	.026	.240
	D2	.333	.680	.126	.059
	D3R	-.014	.320	.006	.792
	D4	.491	.591	.083	-.020
	D5	.080	.544	.194	-.173
	D6	.107	.802	.122	.164

Note: R = reversed items.

**Table 3:** Component transformation matrix.

Component	1	2	3	4
1	.73	.62	.24	.13
2	-.52	.29	.81	-.02
3	.44	-.72	.54	.00
4	-.10	-.11	-.02	.99

### 3.2 Confirmatory Factor Analysis (CFA)

**Figure 1:**
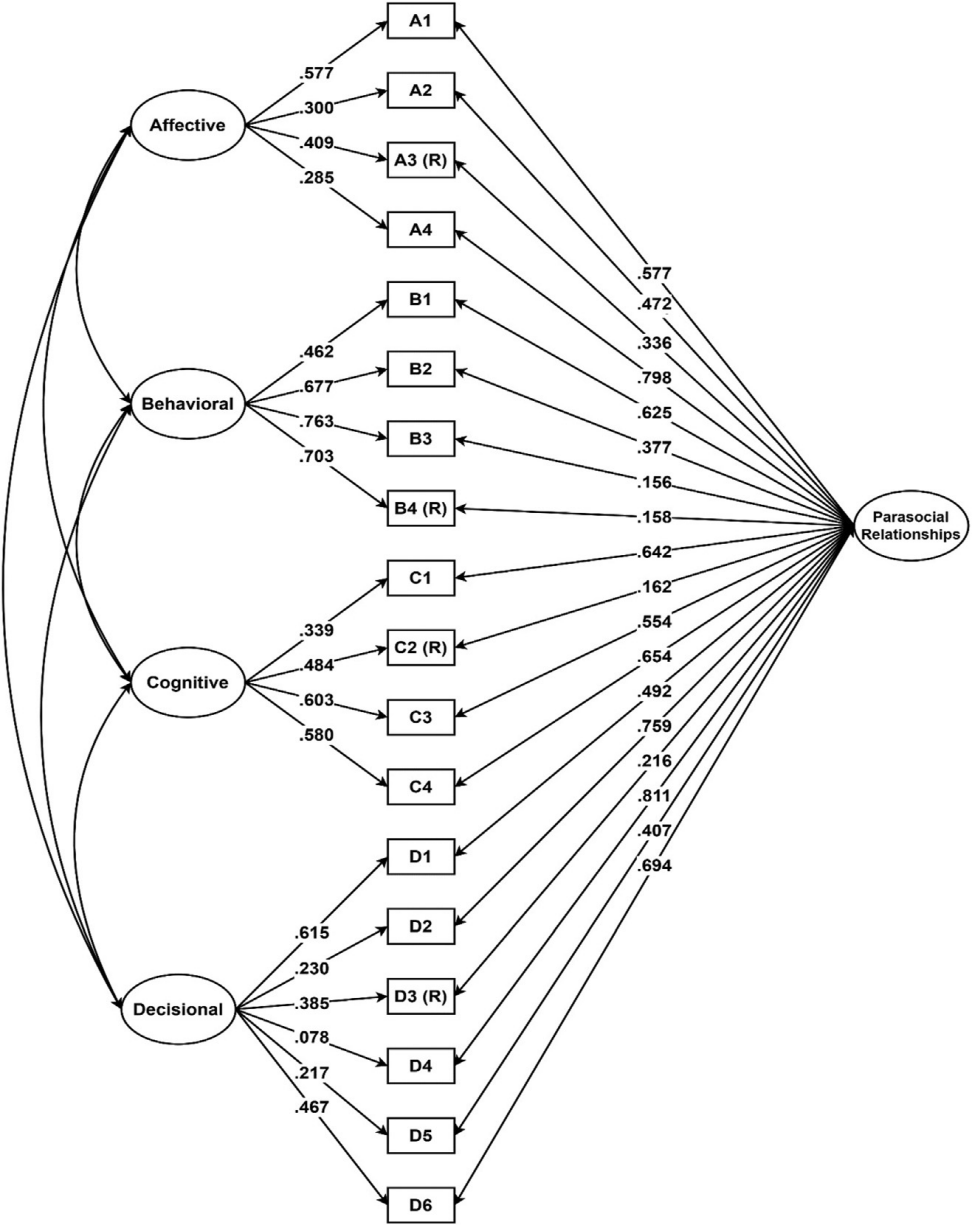
Path diagram for the bifactor correlated factors model of the Multidimensional Measure of Parasocial Relationships and its four dimensions. Note: R = reversed items.

**Figure 2:**
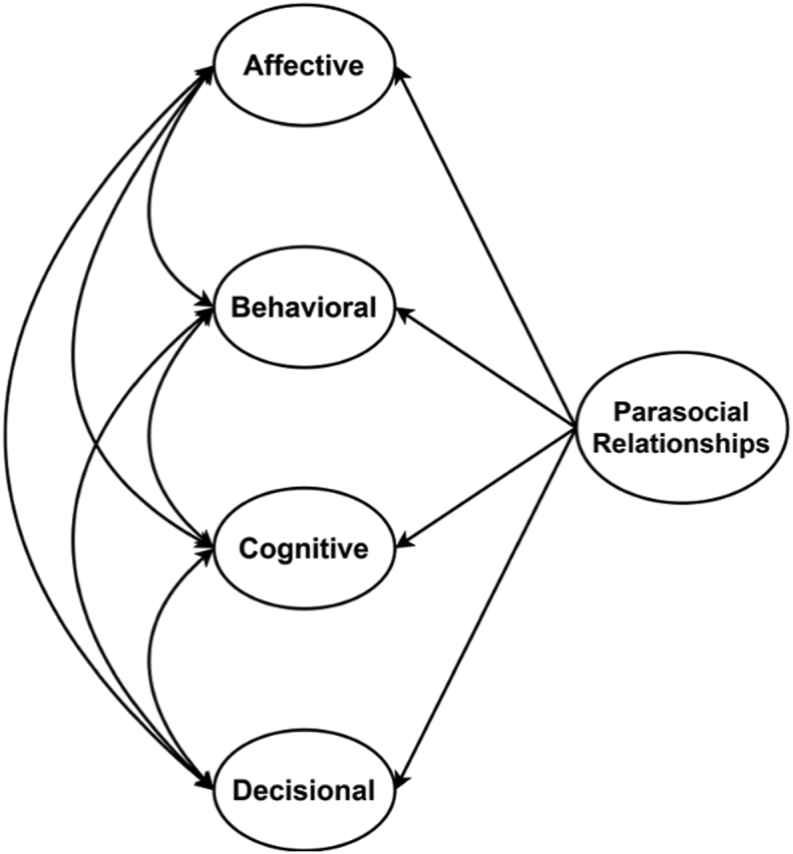
Schematic model of the Multidimensional Measure of Parasocial Relationships (MMPR).

We conducted a CFA in Mplus v7.4 using the Weighted Least Square – Mean and Variance estimation method since the data was ordinal [19]. We tested four models: first-order, second-order, bifactor with uncorrelated factors, and bifactor with correlated factors. Table 4 displays the fit indices for all these four models. The results suggested that the bifactor with correlated factors model had the best fit to the data (Figure 1). Nevertheless, Item D4 had low regression loading, thus, it might need revision and modifications. Figure 2 shows the basic details of the model obtained in this study.

**Table 4:** Fit indices for the factorial models.

Models	Chi-Square	df	p-value	CFI/TLI	RMSEA
First order	378.177	129	.000	.91/.89	.086
Second order	386.757	131	.000	.90/.89	.087
Bifactor (uncorrelated factors)	313.878	119	.000	.93/.91	.080
Bifactor (correlated factors)	241.867	113	.000	.95/.93	.066

### 3.3 Internal consistency

*Cronbach’s alpha* and *ordinal alpha* were calculated to measure internal consistency of the MMPR-dimensions and the total score for the whole measure. *Cronbach’s alpha* and *ordinal alpha* were .71 and .90 for the Affective dimension, respectively; .66 and .95 for the Behavioral dimension, 72 and .90 for the Cognitive dimension, .75 and .88 for the Decisional dimension, and 85 and .93 for the total MMPR-score. Additionally, Guttman split-half coefficient was calculated equal to .82. The correlation within the MMPR dimensions was between .16 and .60, hence showing significant positive relationships that were still distinctive parts of the MMPR (see Table 5).

**Table 5:** Pearson correlation between dimensions and total MMPR-score, *Cronbach’s Alphas*, *means*, and *standard deviations*.

Affective	Affective	Cognitive	Behavioral	Decisional	MMPR
**Cognitive**	.60∗∗				
**Behavioral**	.29∗∗	.16∗∗			
**Decisional**	.57∗∗	.40∗∗	.35∗∗		
**MMPR**	.84∗∗	.75∗∗	.58∗∗	.79∗∗	
***Cronbach’s Alpha***	.71	.72	.66	.75	.85
***Means* and *Sd*. (±)**	2.78 ± 0.64	3.02 ± 0.68	1.58 ± 0.56	2.27 ± 0.64	9.66 ± 1.87

∗∗*p* < .01; MMPR = Multidimensional Measure of Parasocial Relationships; *Sd.* = *standard deviation*.

### 3.4 Mediation analysis

**Figure 3:**
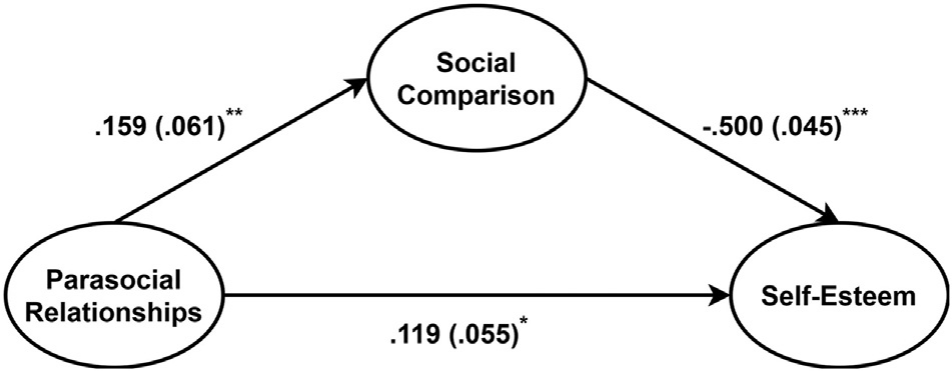
Beta coefficients for all variables in the mediation model in which level of commitment to parasocial relationships (i.e., total MMPR-score) is the independent variable, self-esteem is the outcome, and social comparison is the mediator. Note: ∗p < .05, ∗∗p < .01, ∗∗∗p < .001. Values in parentheses shows the standard errors. MMPR = Multidimensional Measure of Parasocial Relationships.

In the last analysis, we investigated if social comparison mediated the relationship between commitment to parasocial relationships (i.e., the total MMPR-score) and self-esteem. The results showed that the indirect effect of the total MMPR-score on self-esteem was negative and significant (*β* = -.08, *p* < .01). Accordingly, the total MMPR-score by itself (without social comparison) had no negative effect on self-esteem (*β* = .12, *p* < .05), and it was significantly positively related to social comparison (*β* = .16, *p* < .01). Moreover, social comparison was negatively and significantly related to self-esteem (*β* = -.50, *p* < .001). Furthermore, we investigated the significance of the indirect effect of the total MMPR-score on self-esteem using the interactive calculation tool provided by Preacher and Leonardelli [20]. All three tests, Sobel [21], Goodman [22], and Aroian [23], resulted in *p*-values equal to .01. Thus, high levels of commitment to parasocial relationships seem to lead to lower self-esteem through its positive relation to social comparison (Figure 3).

## 4 Discussion

Our aim in this pilot study was to investigate the psychometric properties of the MMPR, a new measure we developed to operationalize how and to what extent people are committed to parasocial relationships. Besides testing the factor structure of the MMPR and its internal consistency, we also investigated the MMPR’s concurrent validity by testing the hypothesized relationship between commitment to parasocial relationships, social comparison, and self-esteem—that is, if people’s tendency to compare themselves to others mediates the relationship between their commitment to a parasocial relationships and their own self-esteem.

All the models we tested showed good fit indexes, specially the bifactor model with four correlated dimensions. The four dimensions and the whole scale showed good internal consistency. Hence, as expected, being committed to a parasocial relationship through social media platforms comprises how people feel (A: Affective), behave (B: Behavioral), and think (C: Cognitive) regarding the media figure, and also to what extent people perceive that their daily decisions (D: Decisional) are influenced by the media figure’s social media activity. The fact that the bifactor correlated model showed the best fit implies that the four dimensions are independent and inter-related, that is, both are at the same time part of one single phenomenon that cannot be measure without all four dimensions. In other words, suggesting that, not only is the MMPR a valid and reliable tool, but also that commitment to parasocial relationships through media platforms consists of these four dimensions (A: Affective, B: Behavioral, C: Cognitive, and D: Decisional).

Moreover, as hypothesized in past research [3, 4, 5, 6], high levels of commitment to parasocial relationships lead to lower self-esteem through its positive relation to social comparison. Indeed, some studies have found that lower self-esteem had a significant positive influence on parasocial relationships [24], while others indicate that high levels of social comparisons were associated with decreased self-esteem [25]. In addition, high levels of commitment to parasocial relationships are associated to both social comparison and lower body satisfaction [26]. Hence, the results in this pilot study are in line with past research, which verifies the MMPR’s concurrent validity. In addition, we have also verified its validity by showing how commitment to parasocial relationships interact with social comparison and self-esteem.

The MMPR is an important addition to the different measures of parasocial relationships and interactions and a unique measure of people’s commitment to parasocial relationships to media figures on social media platforms. Indeed, rather than targeting social media, current measures address people’s interaction with, for example, TV celebrities in the context of the actual TV show or movie [7, 8]—social media platforms are not only the most spread medium for parasocial interactions and relationships with media figures and celebrities, but also permit a unique one-to-one and real-time opportunity to interact with media figures and celebrities through the use of functions such as “Likes” and “Comments”. Moreover, most of the current measures address either parasocial interactions [2, 7, 8] or parasocial relationships [9]; the MMPR address both by considering that these two phenomena are distinct but also related—people can have a parasocial interaction without having a parasocial relationship and vice versa [10], but repeated parasocial interactions with a media figure probably increase the sense of having a parasocial relationship with the media figure [11].

More importantly, to the best of our knowledge, the MMPR is the only measure of parasocial interactions and relationships that has its theoretical basis on the multidimensional model of attitude [13] and on to what extent such attitude is perceived to influence daily life decisions. We argue that this multidimensional structure of commitment to parasocial relationships as a unified phenomenon, might help us to better understand how and why social media figures influence our feelings, thoughts, behaviors, and decisions. We find this important since parasocial relationships and social media have both positive [27, 28] and negative effects on mental health [29, 30, 31, 32], physical health [33, 34, 35], and cultural norms [2]. For instance, social interactions generally accompany commitment to social norms [2, 36], therefore, targeting the four dimensions of commitment to parasocial relationships by the acculturalization and enhancement of internal values through social media might help to address the low levels of self-esteem accompanied by such relationships and interactions.

### 4.1 Limitations

The fact that the present study is solely based on self-reports implies that any causation or generalization statements need to be interpreted with caution and our results need to be replicated in different populations and using different methods (e.g., Item Response Theory). Another main limitation in our pilot study is that we had a convenient sample with limited age range (*mean* = 25 years, *SD* = ±5.15), thus, the MMPR’s psychometric properties in older samples needs still to be tested. Moreover, due to the gender distribution in our convenient sample (207 women, 51 men, 1 other), we were not able to investigate the effect of gender in, for example, the MMPR’s factor structure. This is important for future studies, after all the effect of commitment to parasocial relationships on self-esteem is more accentuated among young females [5, 6].

### 4.2 Conclusion and final remarks

In short, the MMPR has sound psychometric properties (i.e., factor structure, internal consistency, and concurrent validity) and is a good candidate for further analyses and for the measurement of people’s commitment to parasocial relationships with social media figures. At a more general level, the reason behind why we look for role models in social media figures and allow them to influence our feelings, thoughts, behaviors, and daily life decisions is beyond the scope of our pilot study. What we can be certain of, is that the influence from celebrities and figures through social media seems to be here to stay. We argue that a first step to prevent and treat any negative effects of such influence on people’s mental health is to develop methods that measure the nature of this multidimensional phenomena. In this endeavor, we agree in the fact that there is a distinction between relationships and interactions as concepts; but we argue that, especially for social media platforms, both constructs form one single phenomenon that we refer to as commitment to parasocial relationships.

One way of describing and understand our findings is that if people increase their activity in any of the dimensions, for example liking or commenting more in social media (i.e., behavioral dimension), their feelings and thoughts regarding the media figure will also increase and their daily life decisions will be more in line with the media figure’s, leading to higher commitment to the parasocial relationship. This in turn might lead to a higher tendency to compare oneself to others, which will be detrimental for self-esteem. However, this might also depend on the type of media figure one is committed to. Hence, future studies might need to address this fact and how the different dimensions interact to influence people’s self-esteem and social comparison. Although beyond the scope of our pilot study, it is plausible to suggest that the behavioral dimension might be the motor within the commitment to the parasocial relationship. Indeed, when it comes to changes in attitudes, the best way to change the emotions and thoughts towards an object or person, is probably through changes in behavior [37].

"*The world changes by your example, not by your opinion.*"

*Paulo Coelho*

## Declarations

### Author contribution statement

Danilo Garcia, PhD: Conceived and designed the experiments; Analyzed and interpreted the data; Wrote the paper.

Elina Björk: Conceived and designed the experiments; Performed the experiments; Wrote the paper.

Maryam Kazemitabar: Analyzed and interpreted the data; Contributed reagents, materials, analysis tools, Wrote the paper.

### Funding statement

This research did not receive any specific grant from funding agencies in the public, commercial, or not-for-profit sectors.

### Data availability statement

Data will be made available on request.

### Declaration of interest’s statement

The authors declare no conflict of interest.

### Additional information

No additional information is available for this paper.
